# Robust Functionality and Regulation of Selectively Expressed RNA as AAV Vectors and In Vitro Transcribed Molecules

**DOI:** 10.3390/pharmaceutics17121595

**Published:** 2025-12-10

**Authors:** Frederik Rastfeld, Nils Hersch, Georg Dreissen, Hajaani Manoharan, Laura Wagner, Lukas Lövenich, Elke Barczak, Hildegard Büning, Rudolf Merkel, Bernd Hoffmann

**Affiliations:** 1 Institute of Biological Information Processing, IBI-2: Mechanobiology, Research Centre Juelich, 52425 Juelich, Germany; f.rastfeld@fz-juelich.de (F.R.); n.hersch@fz-juelich.de (N.H.); g.dreissen@fz-juelich.de (G.D.); h.manoharan@fz-juelich.de (H.M.); l.wagner@fz-juelich.de (L.W.); l.loevenich@fz-juelich.de (L.L.); r.merkel@fz-juelich.de (R.M.); 2 Institute of Experimental Hematology, Hannover Medical School, 30625 Hannover, Germany; barczak.elke@mh-hannover.de (E.B.); buening.hildegard@mh-hannover.de (H.B.)

**Keywords:** seRNA, cell targeting, expression control, minicircle, IRES, RNA cap, IVT-RNA

## Abstract

**Background/Objectives:** Selectively expressible RNA (seRNA) molecules represent a promising new platform for the induction of cell type-specific protein expression. Based on the sense–antisense interaction of the seRNA antisense domain with target cell-specific RNA molecules, the partial degradation of the seRNA molecule induces the activation of an internal ribosomal entry site to initiate translation. The selective expression of seRNA encoded proteins exclusively in target cells works both in vitro and in vivo but is associated with a lower expression intensity compared with classical mRNAs. Furthermore, seRNAs have so far been transfected into cells by plasmid-encoded seRNA expression systems, which is limiting their broad medical applicability. Here, we focus on the characterization of plasmid-based seRNA uptake and activation as well as on options to transfer the seRNA technology to additional vector systems to increase target cell-specific effector expression. **Methods:** seRNA constructs were generated as expression plasmids, AAV, DNA minicircles and IVT-RNA and delivered into different eukaryotic cell lines by transfection/transduction. Analyses were performed using fluorescence microscopy and, for quantitative analyses, flow cytometry. RNA stability and expression analyses were performed using qRT-PCR. **Results:** We show that seRNA-based plasmid systems are efficiently transfected into cells but that reduced RNA steady-state levels are present compared with control expression plasmids. This effect is most likely based on reduced transcription efficiency rather than seRNA stability. Furthermore, seRNA transcription from viral vectors or circular DNA significantly increased the effector expression of seRNAs and enabled linear expression regulation while maintaining target cell-specific activation and inactivation in non-target cells. Optimal results were achieved by adapting the technology to in vitro transcribed seRNA. **Conclusions:** Our data show that seRNA technology develops its full functionality regardless of the type of transfer vector used. Furthermore, expression strength can be regulated within a wide range while maintaining consistent functionality which will enable broad applicability in medicine in the future.

## 1. Introduction

Developing effective and selective treatment approaches is the holy grail in modern medicine. While cytostatic drugs are highly potent for inhibiting cancer cell growth in vitro, severe side effects are harming patient’s healthy tissues in vivo. In contrast, targeted approaches like CAR-T cell, antibody or antibody drug conjugates (ADCs) are more selective but extremely cost-intensive to manufacture and applicable only to a certain number of cancer types, limiting their clinical efficacy, e.g., for solid tumors [1].

Nevertheless, all these approaches can only target circulating proteins or cell surface markers, from which only about 15% are described as “druggable” and even fewer are validated drug targets to date [2], demonstrating the necessity for new treatment modalities.

Due to the success of the COVID-19 mRNA vaccines during the pandemic in 2021, RNA-based therapeutic approaches experienced a new boost. Besides mRNA approaches, various other types of RNA molecules like siRNAs, aptamers or antisense oligonucleotides can also target diverse intracellular molecules during several steps of the expression and splicing of protein-coding and non-coding genes and RNAs [3]. Consequently, RNA-based approaches open the modality of intracellular disease targets for new therapeutic approaches.

In recent years, novel RNA techniques have been developed to induce the selective expression of mRNA encoded proteins only in specific cell types through different mechanisms, addressing the hurdle of selectivity. Included are eukaryotic toehold switches (eToehold) [4], microRNA-responsive ON and OFF switches [5], the adenosine deaminases acting on RNA (ADAR) technology [6] and, as the latest development, selectively expressed RNA (seRNA) technology [7].

This seRNA enables, as a modular platform, the selective expression of an effector sequence in target cells through the presence of a target cell-specific RNA, while remaining inactive in cells lacking the target RNA. The underlying mechanism is facilitated by the de novo assembly of various highly conserved RNA-based regulatory domains and is also presented in more detail in Supplementary Figure S1. In short, seRNA translation is not induced by ribosomes that bind to the 5′-cap structure (removed from the seRNA by small upstream open reading frames), but by a viral internal ribosomal entry site (IRES). While the IRES is blocked by an IRES-blocking sequence that is interfering with the IRES secondary structure in the seRNA full-length pro-drug form in non-target cells, the IRES blocker is degraded by dsRNA-recognizing RNases. These RNases recognize the sense–antisense interaction of a target cell-specific RNA molecule with a fitting antisense domain of the seRNA. However, complete degradation is blocked by a XrnI RNase-inhibiting secondary structure (stem loop) placed between the IRES blocker and the IRES domains. Consequently, activation in target cells takes place by IRES blocker removal and therefore the induction of the IRES-dependent translation of the effector sequence in target cells only [7]. While such IRES-dependent translation is naturally weaker than m^7^G-Cap-induced translation [8,9], it is currently still unclear if additional mechanisms might have an additional lowering effect on seRNA expression which might impair their medical and biotechnological applicability.

For this reason, in this paper we are analyzing the underlying mechanisms that affect and regulate seRNA expression. Analyses include experiments to enhance seRNA expression intensity without impairing selectivity to improve the seRNA platform technology. Furthermore, since regulatory mechanisms can largely depend on the vector types used, we compare and functionally characterize the already-established pDNA-based seRNA expression with viral expression systems and in vitro transcribed (IVT) seRNA molecules.

## 2. Methods

### 2.1. Plasmid Constructs

All seRNA plasmids used here have been described before [7]. For construction of the α-actinin-eGFP plasmid, non-muscle α-actinin sequence (ca. 2.800 bp, provided by Dr. D. J. Kwiatkowski, Harvard Medical School) was cloned into the pEGFP-N1 vector (4.733 bp, Clontech, San Jose, CA, USA), as described here [7]. Maps of the constructed plasmids are also shown in Supplementary Figure S2.

### 2.2. Cell Culture

All cell types were cultivated at 37 °C in a humidified atmosphere of 5% CO_2_. U87MG glioblastoma cells (ATCCs) were cultivated in MEM medium (Sigma Aldrich, Burlington, MA, USA) supplemented with 10% FBS, 1× PenStrep, 1× NEAA and 1× L-Glutamine (all ThermoFisher, Waltham, MA, USA). Human foreskin fibroblasts (HFFs, ATCCs) were cultivated in DMEM GM medium (ThermoFisher, Waltham, MA, USA) supplemented with 10% FBS and 1× PenStrep (all ThermoFisher, Waltham, MA, USA). Cells were seeded 24 h before treatment on 24-well plates (BD Falcon; Franklin Lakes, NJ, USA; Thermo Scientific, Waltham, MA, USA) for microscopy, flow cytometry and qRT-analysis. For all experiments with labeled plasmids, we used 12-well plates with glass bottoms (BD Falcon; Franklin Lakes, NJ, USA; ThermoFisher, Waltham, MA, USA).

### 2.3. Transfection

Lipofectamine 3000 (Invitrogen, ThermoFisher, Waltham, MA, USA) was used for all transfections. For this, 66,000 U87 cells/cm^2^ or 32,000 HFF/cm^2^ were seeded on substrates and coated for 30 min with 0.01% mg/mL human fibronectin (VWR, Radnor, PA, USA) in PBS. The transfection protocol here described as an in vivo protocol has been adapted from the manufacturers’ protocol as described by us earlier [10]. For RNA degradation experiments we inhibited RNA synthesis with 16 µM Actinomycin D (Carl Roth, Karlsruhe, Germany). For studies on transcription activity, we added Trichostatin A to a final concentration of 350 ng/mL (Sigma-Aldrich, Burlington, MA, USA) to the cells or 10 nM Romidepsin (Sigma-Aldrich, Burlington, MA, USA), 1.5 µM MG149 (Sigma-Aldrich, Burlington, MA, USA) or 1.5 µM C646 (Sigma-Aldrich, Burlington, MA, USA) 3 h after transfection as final concentrations in 500 mL media. The TrE (nucleic acid transfection enhancer, InvivoGen, San Diego, CA, USA) was used according to the manufacturer’s protocol. Particle size and zeta potential were measured using a Zetasizer Nano ZS (Malvern Instruments, Malvern, UK). All measurements were performed at room temperature and were repeated at least 3 times. We measured after dilution in purified and sterile filtered water (Milli-Q Gradient A10, Merck Millipore, Burlington, MA, USA and 0.1 µm sterile acrodisk 25 mm syringe filter by VWR, Radnor, PA, USA).

### 2.4. Microscopy

Microscopy analyses were performed in phase contrast and fluorescence 24 h, 48 h and/or 72 h after treatment at 37 °C and 5% CO_2_ using a confocal laser scanning microscope (cLSM 710, Carl Zeiss Jena, Jena, Germany). The microscope was equipped with a 488 nm argon laser, 633 nm helium–neon laser and a 405 nm laser with appropriate filter settings to visualize eGFP, Cy5 and DAPI. All images of transfected cells showed a representative overview at the center of each substrate. Micrographs were recorded with an EC Plan-Neofluar 10×/0.3 Ph1 objective (Carl Zeiss Jena, Jena, Germany). For the experiments with labeled plasmids a Plan-Apochromat 20×/0.8 Ph2 objective (Carl Zeiss Jena, Jena, Germany) was used. Microscope settings were kept identical for all experiments.

### 2.5. Flow Cytometry

We used flow cytometry (CytoFLEX S Flow Cytometer, Beckmann Coulter, Brea, CA, USA) to determine transfection efficiency and fluorescence intensity. Briefly, 24 h or 48 h or 72 h after treatment, cells were trypsinized with 0.05% Trypsin EDTA solution (Gibco, Thermo Fisher, Waltham, MA, USA) and centrifuged at 200× *g*. Cells were analyzed directly after resuspension in 200 µL of the corresponding culture medium. At least 10,000 cells were analyzed for granularity, size and eGFP fluorescence. For gating of the intact cells, we used a control and gated the untreated cells using the SSC-A and FSC-A channel. From that cell population we measured GFP-positive cells in comparison to an untransfected control using the B525-40 A filter and a gain of 40. The percentage of GFP-positive cells and the mean intensity of the GFP-positive cells were analyzed [7].

### 2.6. Plasmid Labeling

For plasmid labeling we used the Label IT Tracker Intracellular Nuclei Acid Localization Kit, Cy5 (Mirus, Madison, WI, USA) according to the manufacturer’s protocol. We applied a plasmid/tracker ratio of 0.5:1. Before microscopy analysis of the living cells, cells were washed 3 times with 20 u/mL Heparin in PBS to remove lipoplex particles on cell surfaces [11]. Nuclei of living cells were stained using NucBlue Live ReadyProbes Reagent (Hoechst, ThermoFisher, Waltham, MA, USA).

### 2.7. Labeled Plasmid Quantification

To quantify the red signal in “green” and “not green” labeled cells, an in-house developed program was used.

As the first step, a mask of the intense red Cy5 signal was created by smoothing the signal stack smoothed using a Gaussian filter (sigma[x,y] = 3, sigma[z] = 0.5). Then each slice was segmented separately using a local median filter (disk structuring element of radius 3 pixel). Additionally, only pixels exceeding at least 90% of the maximum intensity of the whole red signal stack were tolerated. Finally, on each red signal slice mask, morphological operations (opening with a disk of radius 2 pixel; removing objects smaller than 35 pixels; closing holes smaller than 35 pixel) were performed.

Next, nuclei were detected in the nuclei image stack. Here also a Gaussian filter (sigma[x,y] = 1) was used and subsequently a maximum intensity projection (MIP) was performed. Then a threshold using Otsu’s method [12] was used to segment the nuclei from the background. Nuclei with a size of less than 100 pixels were discarded and connected nuclei were separated using the watershed algorithm.

Then the intensity of the red labeled signal within the nuclei was analyzed in various slices. For this purpose, the MIP of the nuclei image was used again. Then for each x- and y-position, the z-slice index of the maximum intensity was saved instead of the maximum intensity itself. Then, individually for each nucleus, the median of the z-slice indices was calculated. This z-index ± 3 slices was then used to analyze the red labeled signal within each nucleus.

To separate the results into two groups, “eGFP-expressing cells” and “not eGFP-expressing cells”, the eGFP image stack was used. Again, the MIP was calculated on the green image stack and Otsu’s method was used as a threshold to separate signal and background. To be more sensitive, the threshold was multiplied by 0.5 but had to be at least 50 (within a gray value range from 0 to 255). If a previously detected nuclei was covered by at least 50% of this green labeled signal, this cell/nucleus was defined as a “green cell”, otherwise as a “not green cell”.

Additionally whole cell shapes were drawn manually, labeled as “eGFP-expressing cells” or “not eGFP-expressing cells” and analyzed in the same manner. Here again, the median z-slice indices of the nuclei were used to define the z-slices that were analyzed.

### 2.8. qRT-PCR

For RNA quantification, total RNA was isolated using the RNeasy Plus Mini Kit (QIAGEN, Hilden, Germany) in combination with QIAshredder columns (QIAGEN, Hilden, Germany). We performed the cDNA synthesis using a QuantiTect Reverse Transcription Kit (QIAGEN, Hilden, Germany). cDNAs of interest were quantified using 0.1 µg of total cDNA using a StepOne Real-Time PCR System (Applied Biosciences, Thermo Fisher, Waltham, MA, USA) and TaqMan assay-specific primers (Table 1) and master mix (Applied Biosciences, Thermo Fisher, Waltham, MA, USA). As the endogenous control, we used glyceraldehyde 3-phosphate dehydrogenase (GAPDH, Thermo Fisher, Waltham, MA, USA). For evaluation, we used StepOne Software (Version 2.0.2).

For DNA quantification, total DNA was isolated using a QIAamp DNA Mini Kit (QIAGEN, Hilden, Germany). Isolated DNA was quantified as described for cDNA above. For calculation of DNA amounts, a serial dilution from each plasmid was analyzed using qRT-PCR to generate a straight calibration line.

### 2.9. Nuclei Isolation

Subcellular fractionation was performed as described before [13]. Briefly, cells were washed with PBS and media to remove any membrane-bound vector particles. A Subcellular Protein Fractionation Kit for Cultured Cells (Thermo Fisher, Waltham, MA, USA) was used to isolate the nuclear fraction. From this fraction DNA was isolated as described above.

### 2.10. AAVs

AAV vectors were produced in HEK293 by triple transfection of AAV helper plasmid pRC’99-VSSTSPR [14] the vector genome plasmid of seRNA_Δ3-5_-eGFP or seRNA-eGFP, and the adenoviral helper plasmid pXX6 (PMC109519) as described [15]. The vector genome plasmid encodes for the respective seRNA constructs in a self-complementary vector genome configuration [14]. After 48 h transfection, cells were harvested, lysed by thaw–freezing cycles and lysate was cleared by Benzonase treatment and low-speed centrifugation. Pre-cleared lysate was purified by iodixananol strep gradient ultracentrifugation, harvesting the vector-contained 40% phase as described [15]. Vector preparations were re-buffered by heparin affinity chromatography followed by a dialysis step.

### 2.11. Minicircles

We obtained the seRNA-Caspase3ds minicircle from Plasmid Factory (Bielefeld, Germany). The concentration and buffer were equivalent to the other plasmids used. Cell viability was measured using a CellTiter-Glo 2.0 Assay (Promega, Madison, WI, USA) according to the manufacturer’s protocol. For readout of the luminescence we used Tecan i-control, 2.0.10.0, infinite M1000Pro (Tecan, Männedorf, Switzerland).

### 2.12. In Vitro RNA Synthesis

All eGFP and seRNA sequences were cloned into a pBluescript SK-vector. We linearized plasmids and used a T7 MegaScript Kit (Themo Fisher, Waltham, MA, USA) for in vitro transcription (IVT). As cap analogs we used m7GP3G, AP3G and GP3G (all Jena Biosciences, Jena, Germany). RNA purification was performed using LiCl Solution (Thermo Fisher, Waltham, MA, USA). We verified the purity of each newly synthesized IVT-RNA by denaturing 1% agarose gel using electrophoresis (1.5 h at 100 V). An example is shown in Supplementary Figure S3. We transfected the RNAs using Lipofectamine3000 (Thermo Fisher, Waltham, MA, USA) according to the manufacturer’s protocol without the use of P3000 reagent.

### 2.13. Statistical Analysis

All data are given as a mean including standard deviation. We performed at least three independent measurements for each experiment. Statistical analysis (univariate ANOVA) was performed using Microsoft Excel (Microsoft Office Standard 2016, Microsoft, Redmond, WA, USA) for multiple comparisons. Graphs were created using Origin 2019 32 Bit (Version 9.6.0.172) (Origin Lab Graphing & Analysis). A *p*-value of ≤0.05 was considered as significant, *p*-values < 0.05, <0.01 and <0.001 were labeled with one to three asterisks, respectively. For image composition we used ImageJ 1.53f51 (National Institutes of Health, Bethesda, MD, USA) and Microsoft PowerPoint (Microsoft Office Standard 2016, Microsoft, Redmond, WA, USA).

## 3. Results

### 3.1. seRNA Molecules Specifically Target Pre-Chosen Cell Types

**Figure 1:**
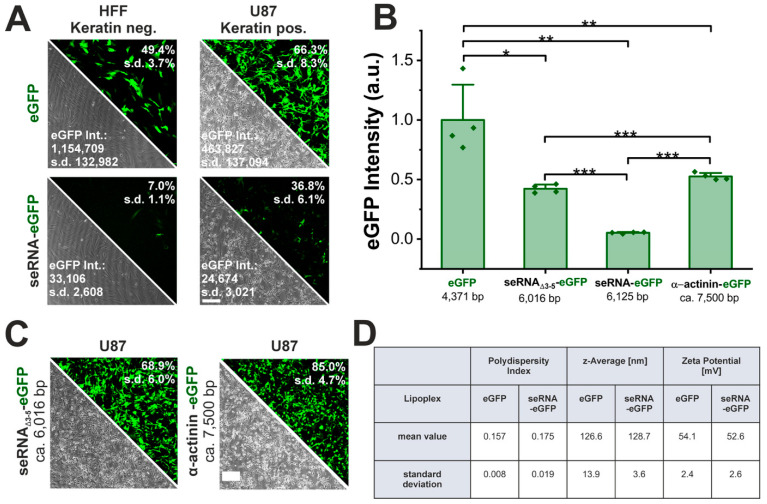
Independency of seRNA expression on lipoplex characteristics and plasmid size. (**A**) Functionality of the seRNA-eGFP plasmid in comparison to a conventional eGFP plasmid after transfection in human foreskin fibroblasts as non-target cells and U87 glioblastoma target cells 24 h post transfection. Note that in all cases eGFP intensity values are exclusively based on the eGFP-positive cell fraction. Scale bar: 200 µm. n = 4 independent samples. (**B**,**C**) Comparison of transfection efficiency and expression intensities (mean fluorescent intensity) of seRNA and control plasmids with varying size and sequence 24 h post transfection. Scale bar: 200 µm. n = 4 independent samples. (**D**) Analysis of the polydispersity index, z-average and zeta potential maxima as determined by DLS of the eGFP and seRNA-eGFP Lipofectamine3000 LNP. n = 3 independent samples. Mean values with s.d. are given. *p*-values were calculated using a one-sided ANOVA test. *p*-values of 0.05, 0.01 and 0.001 are indicated with one to three *.

seRNA molecules are characterized by target cell-specific expression with low leakiness in non-target cells with an additionally reduced expression intensity. To verify the functionality of seRNA molecules, a seRNA expression plasmid comprising a keratin13 motif as the antisense sequence was employed to specifically target keratin-expressing glioblastoma cells (U87) and to induce the IRES-dependent translation of eGFP as the effector. Human foreskin fibroblasts (HFFs) lacking keratin served as negative control cells. As the unselective control, a conventional eGFP plasmid was transfected and showed 49% eGFP-positive HFFs and 66% positive U87 cells, which were high transfection efficiencies for both cell types (Figure 1A). In contrast, the seRNA-eGFP plasmid demonstrated stable eGFP expression levels only in U87 target cells (37%), while eGFP expression was strongly reduced in HFF non-target cells (7%).

Even though seRNA constructs proved their target cell-specific translational activation in target as well as in non-target cells, overall expression intensities were reduced by 95% in target cells (from 463,827 to 24,674) and even more so in non-target cells (97%, from 1,154,709 to 33,106) (Figure 1A,B).

To understand the underlying cause of reduction in transgene expression level compared with non-seRNA constructs, we first analyzed the formed lipoplexes, focusing on their polydispersity index (PDI), size (z-average) and surface charge (zeta potential) and could not observe differences between the conventional eGFP plasmid and the seRNA eGFP plasmid (Figure 1D). Since seRNA plasmids have an extended length due to their domain structure, we next examined the influence of plasmid size. Here, a control plasmid of a comparable length and similar sequence as the seRNA-eGFP plasmid (seRNA_Δ3-5_-eGFP: seRNA construct w/o antisense, IRES blocker and stem loop) or even an enhanced length (α-actinin-eGFP) were effectively expressed at an approximately 10-fold level compared with seRNA-eGFP, despite a slight reduction in eGFP expression compared with the eGFP plasmid only (Figure 1B,C). These data also prove the importance of regulatory seRNA domains 3–5 for expression regulation. The data argue that the presence of such domains, and therefore the switch from unselective, CAP-dependent expression to selective, IRES-based expression, is the main reason for the reduced expression intensity of functional seRNA constructs.

### 3.2. seRNA Plasmids Are Effectively Transferred into the Nucleus by LNPs

**Figure 2:**
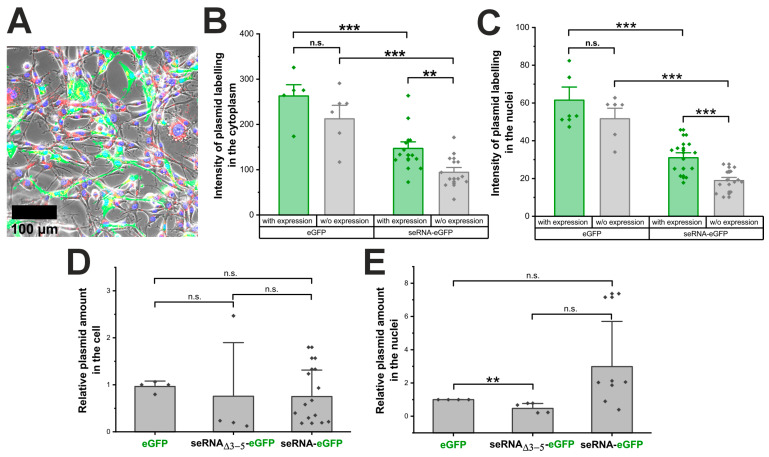
Plasmid transfer is comparable between seRNA and controls. (**A**) Micrographs of Cy5-labeled eGFP plasmids (red) coding for eGFP (green), delivered in U87 glioblastoma cells with nuclei stained in blue. Scale bar: 100 µm. For better visibility, separate channels are also given in Supplementary Figure S4. (**B**) Analysis of the Cy5 cytoplasmic signal of the labeled eGFP plasmid and the seRNA-eGFP plasmid in eGFP-positive cells 24 h after transfection. (**C**) Intensity from the labeled eGFP plasmid and seRNA-eGFP plasmid in isolated cell nuclei. (**D**) qRT-PCR quantification of the eGFP, control seRNA_Δ3-5_-eGFP and the seRNA-eGFP plasmid in U87 cells. (**E**) Relative quantification of the three plasmids only in the nuclei of the transfected cells. All quantifications were performed 24 h post transfection and were based on 3D stacks for optimal detection of plasmid localization. All *p*-values were calculated using a one-sided ANOVA test. Data are presented as mean values with s.d. of at least three independent samples. *p*-values of 0.01 and 0.001 are indicated with two to three *. n.s. = not significant.

Besides the switch from classical CAP to IRES-based expression for activated seRNA constructs, additional factors might impact expression levels. Hypothesizing that seRNA plasmids are recognized by lysosomal or cytoplasmic pattern recognition receptors, we first analyzed whether the same quantities of Cy5-labeled seRNA-eGFP and control eGFP plasmids enter the cell and subsequently translocate into the nucleus. Following the labeling during transfection, plasmids remained functional and trackable in live cells (Figure 2A and Figure S4).

The quantification of the Cy5 signal of the eGFP and seRNA-eGFP plasmids in GFP-positive cells showed a significant difference with higher labeling intensities for eGFP plasmids compared with seRNA-eGFP plasmids. This difference was reproducibly detectable in the cytoplasm as well as for plasmids within the nucleus of GFP-positive cells (Figure 2B,C). We found lower plasmid amounts in cells that did not express eGFP, although this difference was not statistically significant. However, the eGFP plasmid was present in larger amounts in the transfected cells than the seRNA-eGFP plasmid. In a subsequent analysis, the Cy5 plasmid signal was quantified exclusively within the nucleus of the transfected cells to investigate the nuclear delivery of the plasmid. Therefore, we detected larger amounts of both plasmids within the nuclei of eGFP-expressing cells compared with the nuclei of non-eGFP-expressing cells. Once more, the quantity of the eGFP plasmid delivered was statistically significantly higher than that of the transferred seRNA-eGFP plasmid.

To verify these results, we additionally isolated the total DNA of cells that were transfected with eGFP- and seRNA-eGFP plasmids 24 h before isolation. As the control, the non-selective seRNA_Δ3-5_-eGFP construct was also analyzed. Interestingly, all plasmids were detected with comparable amounts (Figure 2D). Although with an enhanced spread of data points, comparable plasmid concentrations for all constructs were also found after the isolation of nuclei and qRT-PCR-based quantification (Figure 2E). These data therefore argue that seRNA plasmids are effectively transferred into the cell and the nucleus without the major recognition of seRNA sequences by innate immune mechanisms upon DNA-based transfection.

### 3.3. seRNA Molecules Are Comparably Stable

**Figure 3:**
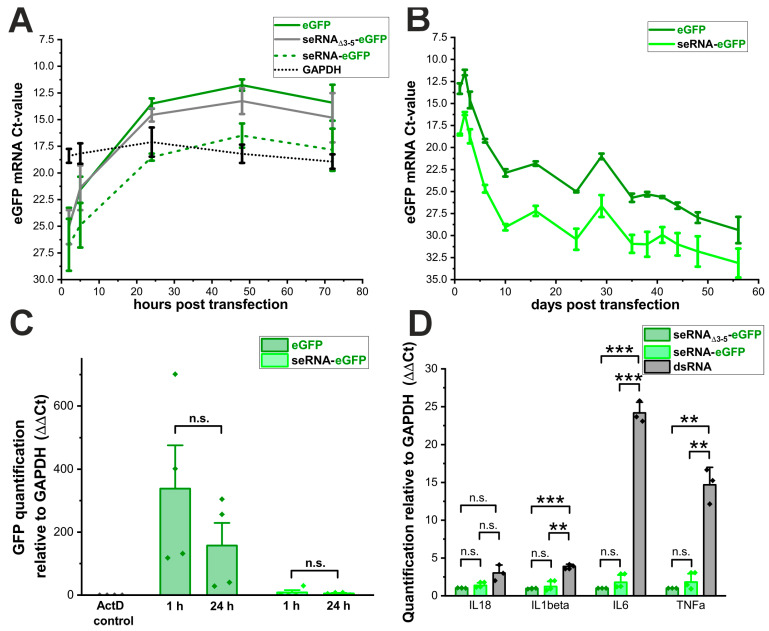
Basic seRNA plasmid transcription is reduced. (**A**) Analysis of the RNA expression: Ct-values from the eGFP plasmid, control seRNA_Δ3-5_-eGFP plasmid and the seRNA-eGFP construct after transfection in human U87 glioblastoma cells. RNA expressions were analyzed after 2 h, 5 h, 24 h, 48 h and 72 h. The expression of the housekeeping gene GAPDH is shown in black. n = 4 independent samples. (**B**) Degradation analysis of the RNA transcribed from the eGFP plasmid and seRNA-eGFP plasmid 1 h and 24 h after transcription inhibition using Actinomycin D. As the transcription inhibition control, we added 30 min post eGFP plasmid transfection. n = 4 independent samples. *p*-values were calculated using one-sided ANOVA. (**C**) Long term RNA stability analysis after eGFP plasmid and seRNA-eGFP plasmid transfection. n = 3 independent samples. (**D**) Analysis of the immune reaction 24 h post seRNA-eGFP plasmid transfection in comparison to control seRNA_Δ3-5_-eGFP plasmid and a synthetic dsRNA (Poly I:C). n = 3 independent samples. *p*-values were calculated using one-sided ANOVA. Data are presented as mean values of at least three independent samples with error bars representing s.d. *p*-values of 0.01 and 0.001 are indicated with two to three *. n.s. = not significant.

Since the transfection rates for seRNA plasmids were comparable to the controls, we next analyzed the corresponding RNA transcript levels after plasmid transfection into U87 cells over time. Starting from highly comparable low levels for all plasmids 2 h after transfection, eGFP-RNA concentrations went up to similar levels and with a comparable time course for eGFP as well as seRNA_Δ3-5_-GFP plasmids to reach steady-state levels after approximately 24 h (Figure 3A). These high RNA levels remained almost unchanged for both plasmids over the complete analysis time of 72 h. In contrast, GFP-RNA levels after the transfection of seRNA-eGFP plasmids were significantly reduced. Ct-values argue for approximately 8- to 16-fold reduced RNA amounts with a similar steady-state behavior over time for seRNA-eGFP plasmid-dependent transcription as for the control plasmids. Stable GAPDH RNA levels over time served as the internal control and are shown here as a separate curve to prove that even reduced seRNA-eGFP mRNA levels reach steady-state levels above the control.

To verify these results, we additionally performed long-term experiments with eGFP and seRNA-eGFP plasmids. Following the transfection of the plasmids, a portion of the cells was used at each passage to isolate RNA and to quantify the amounts of the respective transcripts. The data confirm a significantly and consistently lower amount of detectable RNA after the transfection of seRNA-eGFP plasmids compared with eGFP plasmids over an analysis period of approximately two months. (Figure 3B). The overall decrease in RNA quantity over time was again comparable for both constructs, indicating that plasmid stability was similar for both constructs.

Reduced RNA levels after the transfection of full-length seRNA encoding plasmids can either be due to reduced transcription or impaired RNA stability. To explore these possibilities, we used Actinomycin D 24 h post DNA transfection to inhibit further RNA synthesis. After 1 h of the 24 h Actinomycin D-treatment, RNA levels were quantified via qRT-PCR to determine the extent of RNA degradation. For the eGFP plasmid, no difference in eGFP RNA amount was observed after 1 h or 24 h of suppressed RNA synthesis (Figure 3C). Following the transfection of the seRNA-eGFP plasmid, we detected a slight decrease in RNA transcript level 24 h after RNA synthesis inhibition. However, the non-significant reduction of approximately one-third of the seRNA amount within 24 h is not equivalent to the massively reduced expression.

Since no significant differences in RNA half-lives could be detected for seRNA-GFP and eGFP mRNAs, we additionally tested if underlying plasmids or resulting RNAs would induce the transcriptional activation of various immune modulators. Therefore, 24 h after the transfection of the seRNA-GFP and the underlying control plasmid seRNA_Δ3-5_-eGFP, we quantified the RNA levels of Interleukin 18 (IL-18), Interleukin 1 beta (IL-1β), Interleukin 6 (IL-6) and TNF alpha (TNFα) by qRT-PCR. Synthetic double-stranded RNA (dsRNA) served as the positive control. Interestingly, despite the underlying seRNA activation mechanism and interaction between target sense RNA with the antisense domain of full length seRNAs, no transcriptional activation of the indicated immune modulators or significant differences between seRNA-eGFP and seRNA_Δ3-5_-eGFP could be detected (Figure 3D).

### 3.4. Substantially Improved seRNA Expression Using AAVs

**Figure 4:**
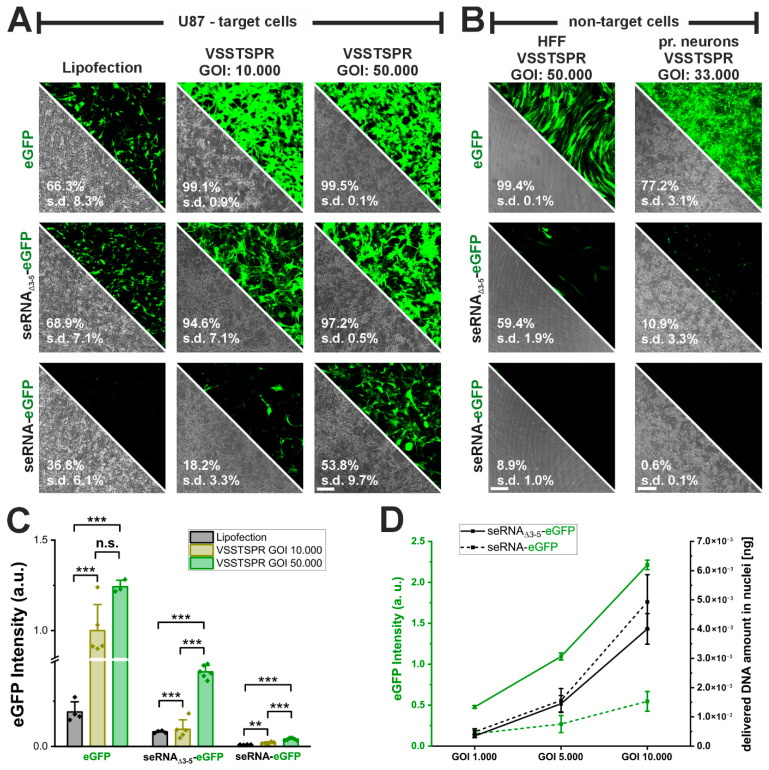
Increase in AAV vector genome transfer equally enhances seRNA expression. (**A**) Comparison of transfection efficiency between lipofection and AAV vectors using VSSTSPR capsid as delivery vehicle for eGFP plasmid, control seRNA_Δ3-5_-eGFP and seRNA-eGFP construct in U87 target cells. n = at least 3 independent samples Scale bar: 200 µm. (**B**). Selectivity test of AAV vectors as delivery vehicle for eGFP plasmid, control seRNA_Δ3-5_-eGFP and seRNA-eGFP construct in HFF non-target cells. n = at least 3 independent samples. Scale bar: 200 µm. All numbers are based on flow cytometry (**C**) Comparison of eGFP expression intensity post delivery of eGFP, control seRNA_Δ3-5_-eGFP and seRNA-eGFP construct via lipofection and AAV vectors. n = at least 3 independent samples. *p*-values were calculated using one-sided ANOVA. Data are presented as mean values with error bars representing s.d. (**D**) Analysis of eGFP expression intensity and delivered DNA amount following application of different genes of interest (GOIs) for delivery of seRNA_Δ3 5_-eGFP construct to the seRNA-eGFP construct n = 3 independent samples. Data are presented as mean values with error bars representing s.d. *p*-values of 0.01 and 0.001 are indicated with two to three *. n.s. = not significant.

Our data show that seRNA-dependent expression levels are lower than for mRNA molecules upon plasmid-based transfection. For this reason, we tested if expression could be increased by adeno-associated virus (AAV) vector-mediated transduction without impairing target cell specificity. Given the size of seRNAs, a self-complementary vector genome configuration was chosen. Moreover, we decided on an engineered AAV serotype 2-derived capsid, VSSTSPR, as this variant is capable of overcoming intracellular barriers. Two distinct particles per cell ratios (genes of interest, GOIs, 10.000 and 50.000) were compared with plasmid transfection. Transduction with both GOIs yielded a percentage of GFP-positive cells, i.e., efficiencies, above 95% for both positive controls (eGFP-AAV and seRNA_Δ3-5_-eGFP-AAV) (Figure 4A). In all cases transduction efficiencies were therefore higher than those found for plasmid transfection. Interestingly, for the full-length seRNA-eGFP construct, transduction efficiencies were found to be lower to those of plasmid transfection with a GOI of 10.000 but could be enhanced three-fold to almost 55% with a GOI of 50.000. Such values were significantly higher than those for plasmid transfection and show the effective scalability upon use of AAV vectors.

To ensure that seRNA target cell specificity was maintained also upon AAV vector use, we tested the percentage of GFP-positive cells in two different non-target cells, i.e., HFF and rat primary cortical neurons with high GOIs. For both positive controls (eGFP-AAV and seRNA_Δ3-5_-eGFP-AAV) we observed a high number (60% to almost 100%) of GFP-positive cells. (Figure 4B) in HFFs. In contrast, for full-length seRNA constructs GFP expression was basically absent in HFF cells with just very low intensities in about 9% of the cells. For rat primary neurons the same selectivity could be identified. However, serotype cell specificity reduced transduction efficiencies here in all cases with the most obvious reductions for seRNA backbone constructs. In summary, the data clearly show that seRNA selectivity remained functional also upon the use of AAVs at high GOIs.

Overall, the use of AAV vectors also resulted in enhanced expression intensities in GFP-positive target cells (Figure 4A,C). Intensities further peaked with increasing GOI to ultimately reach expression intensities also for seRNA full-length constructs (GOI 50.000) that were formerly measured for plasmid-based transfection only for GFP-mRNA control constructs. These data argue for a well-tunable seRNA expression intensity upon the use of AAV vectors. We verified this correlation also for the reduced GOIs of the control seRNA_Δ3-5_-eGFP construct and the seRNA-eGFP AAV. Besides GFP intensity, we additionally determined the number of vector genomes in cell nuclei (Figure 4D). For both constructs we identified a similar and almost linear AAV-DNA increase with increasing GOIs from 1.000 to 10.000. However, while GFP intensities for both constructs also increased with enhancing GOI, the slope of such increase was different with intensities of approximately 1/10 for full-length seRNA constructs compared with the control to reach similar values for seRNA at a GOI of 10.000 that was detected for seRNA_Δ3-5_-eGFP already at a GOI of 1.000.

### 3.5. Positive Effect of Transfection Enhancers on seRNA Expression

**Figure 5:**
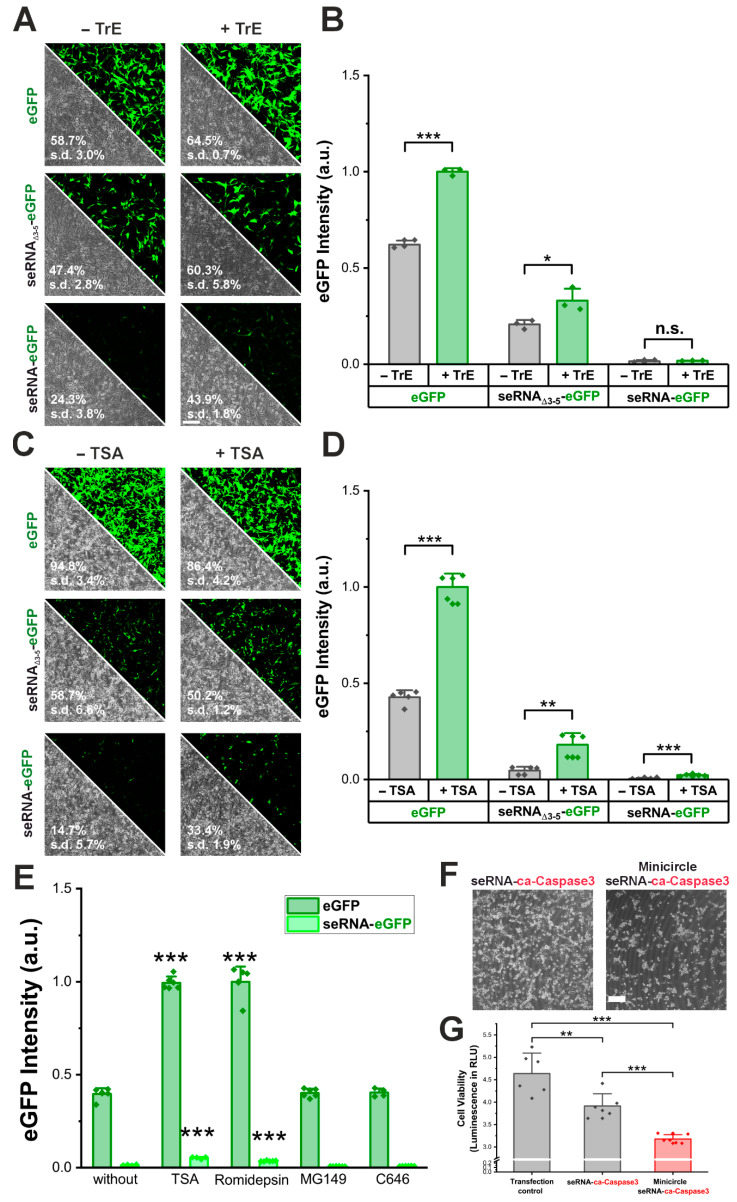
Methods to enhance seRNA expression. (**A**) Microscopic images of U87 glioblastoma cells after transfection of eGFP, seRNA_Δ3-5_-eGFP and seRNA-eGFP plasmid with and without transfection enhancer, (**B**) and the resulting eGFP expression intensity. n = 3 independent samples. *p*-values were calculated using one-sided ANOVA. Scale bar: 200 µm. Data are presented as mean values with error bars representing s.d. (**C**) Microscopic images of U87 cells after transfection with and without Trichostatin A as transcription enhancer, (**D**) and the resulting eGFP expression intensity. n = 5 independent samples. *p*-values were calculated using one-sided ANOVA. Scale bar: 200 µm. Data are presented as mean values with error bars representing s.d. (**E**) Effect of HDAC inhibitors and HAT inhibitors on the transcription of the seRNA-eGFP plasmid and eGFP plasmid. *p*-values were calculated using a one-sided ANOVA comparing values to the respective control without transfection enhancer. Data are presented as mean values with error bars representing s.d. (**F**) Comparison of standard seRNA-ca-Caspase3 plasmid and a seRNA-ca-Caspase3 minicircle plasmid. Scale bar: 200 µm. (**G**) Cell viability 48 h after transfection of eGFP transfection control plasmid, seRNA-ca-Caspase3 plasmid or minicircle seRNA-ca-Caspase3. n = 6 independent samples. *p*-values were calculated using one-sided ANOVA. Data are presented as mean values with error bars representing s.d. All given numbers are based on flow cytometry analyses. *p*-values of 0.05, 0.01 and 0.001 are indicated with one to three *. n.s. = not significant.

AAV vectors mediated enhanced seRNA expression levels and the percentage of positive cells. However, due to their immunogenicity, limitations are present for various medical applications. Therefore, we first tried to increase plasmid-based expression by using a commercial transfection enhancer (TrE). As a result, transfection efficiencies increased for control plasmids (eGFP and seRNA_Δ3-5_-eGFP) as well as full-length seRNA-eGFP. Here, we detected an increase of more than 80% (from 24% to 44% (Figure 5A). While increased transfection efficiencies went along with slightly elevated expression intensities for control plasmids, no such effect was seen for seRNA-eGFP (Figure 5B).

Since the exact composition of TrE remains unknown, we repeated the same analyses using Trichostatin A (TSA), a compound with well-described transfection-enhancing properties. As a result, not only did the transfection efficiency of seRNA-eGFP plasmids more than double (Figure 5C), but the expression intensity also increased almost four-fold (Figure 5D,E). For the additional expression amplifiers, Romidepsin, MG149 and C646, only for Romidepsin could we show a similar increase in expression while the others showed no positive effect (Figure 5E).

Since transfection enhancers are typically not meant to be used for in vivo applications, we additionally tested for seRNA efficacy enhancement by the use of DNA minicircles that are well described for enhanced expression levels due to size reduction [16]. For this purpose, we used a seRNA DNA minicircle that expressed a constitutively active caspase 3 (ca-Caspase3) instead of the eGFP effector, upon transduction in target cells. As a result, killing efficiency increased significantly after 48 h based on ATP cell viability tests with additional major morphological cell surface detachment and rounded cell morphology for most remaining cells (Figure 5F,G).

### 3.6. IVT-seRNA Is Highly Dosable and Allows Precise Modulation

**Figure 6:**
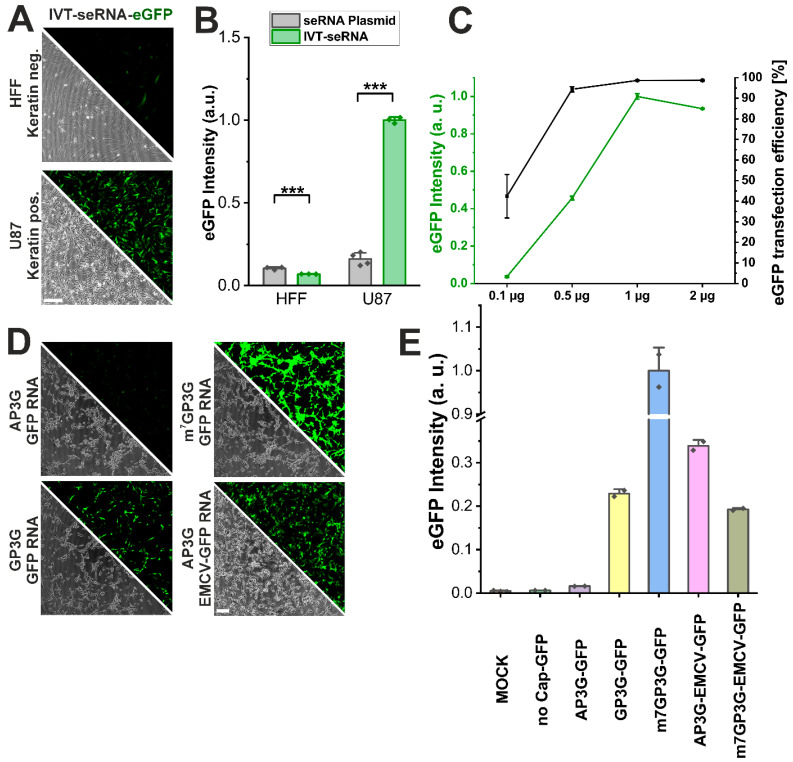
In vitro transcribed seRNA demonstrates high dosability and allows precise modulation. (**A**) Micrographs and (**B**) expression intensities of IVT-seRNA-eGFP in HFF non-target cells and U87 target cells. Scale bar 200 µm. n = 3 independent samples. *p*-values were calculated using one-sided ANOVA. (**C**) IVT-seRNA dose-dependent expression in vitro quantified by eGFP intensity and transfection efficiency in U87 cells. n = 3 independent samples. (**D**) Micrographs of U87 cells transfected with unselective eGFP mRNAs comprising different 5′ cap structures and comprising the EMCV IRES element in combination with AP3G cap structure presented in conjunction with respective eGFP intensity (**E**). n = at least 2 independent samples. Scale bar = 200 µm. *p*-values of 0.001 are indicated with three *.

For medical indications, IVT-RNA would largely expand the applicability of the seRNA technology. For this reason, we generated plasmids for in vitro transcription and tested the resulting IVT-seRNA-eGFP for target cell-specific activation in HFF non-target and U87 target cells, as before. With almost all cells being detectable using flow cytometry and fluorescence microscopy, transfection efficiency as well as target cell-specific activation was significantly higher than that found for pDNA-seRNA transduction. Interestingly, eGFP expression intensity is also more than four-fold higher than for pDNA-seRNA upon IVT-seRNA activation. In contrast, non-target primary cells barely activate IVT-seRNA constructs with baseline expression intensities even lower than those detected after pDNA-seRNA transduction (Figure 6A,B). Increasing IVT-seRNA concentrations additionally show that an almost complete transfection and seRNA activation efficiency is already reached at low concentrations of 0.5 µg per well (125,000 cells in 500 µL). However, expression intensities show an almost linear increase in behavior up to 1 µg to reach a steady-state level at even higher seRNA concentrations without any additional increase (Figure 6C).

seRNA constructs were developed in the first place for pDNA-dependent expression. This resulted in the necessity to block CAP-dependent ribosomal expression via various sequence modifications. Since IVT-seRNA also proved its cell type-specific activation and therefore the robustness of the technology, we additionally tested if CAP-dependent expression could be blocked by the use of CAP molecules that stabilize RNA molecules without interacting with ribosomal subunits. These so-called “silent CAPs” should block the eGFP expression of IVT-eGFP-mRNA completely. With AP3G and GP3G we used two different silent CAPs during in vitro transcription and compared the expression intensities of the resulting mRNAs in U87 target cells with an M7G CAP, used as the positive control. As expected, M7G-mRNA showed the highest GFP expression intensities in almost all cells. In contrast, for AP3G-capped eGFP-mRNA, expression intensities were like those of MOCK-transfected control cells or those transfected with uncapped molecules, and therefore non-functional mRNA. For GP3G, however, expression silencing worked only in parts with intermediate eGFP intensities (Figure 6D,E).

To further verify that AP3G capping was functional, we used the silence CAP for our seRNA_Δ3-5_-eGFP in vitro transcription (AP3G-EMCV-GFP). After transfection, this construct showed expression intensities that were even higher than those detected for the corresponding M7G-EMCV-GFP-positive control and therefore prove the efficient CAP-dependent silencing of AP3G-capped RNA constructs.

## 4. Discussion

The technology of selectively expressed RNA molecules represents an extremely promising approach for therapeutic applications. By combining RNA technology, which can be adapted to a specific cell type, with the possibility of IRES-dependent cell functionalization, this technique achieves cell type-specific protein expression. This breaks new ground by creating new opportunities, in particular, by enabling the activation of seRNA via intracellular markers at the RNA level, thereby complementing therapeutic approaches [17] that are limited to surface markers.

At the same time, however, several questions still need to be clarified regarding this very young technology, of which the aspects of reduced expression intensity, innate immune responses, and the transferability of plasmid-encoded seRNA expression to other vector systems were specifically considered here.

For some therapeutic indications, one challenge of the seRNA technology is the reduced expression of the coding sequence [7]. One of the reasons for this is the need for the IRES-dependent expression of the effector after seRNA activation in target cells. IRESs represent a highly heterogeneous group of secondary structures [18] that are responsible for the regulation of CAP-independent translation in organisms from viruses to humans [19]. Although these secondary structures interact in different ways, mostly with translation initiation factors or ribosomal subunits, the translational activity of an IRES is usually at least an order of magnitude lower than that described for CAP-dependent translation initiation [20]. We find the same here for the EMCV IRES used, even without additional regulatory domains of the seRNA.

However, it is interesting to note the further approximately 10-fold reduction in expression when using a full-length seRNA construct in target cells. At this point, we can only speculate about the reasons. We were able to show that the increased length of a seRNA plasmid and the transfer of the plasmid into the cell nucleus do not play a role in the reduced translation. The latter would theoretically be possible due to the potential recognition of viral sequence motifs used within the seRNA [21]. This recognition would then also be associated with the innate immune response activation [22], which we were able to rule out for seRNAs in both target cells and non-target cells. The analysis performed here on seRNA quantity over time suggests that one reason for the reduced expression is an overall decrease in RNA quantity when transducing seRNA as pDNA-based vectors. Our analyses were unable to conclusively clarify whether less seRNA is transcribed or whether the half-life of seRNA is reduced, although our experiments do not indicate any change in seRNA stability. In addition, it is well established that pDNA can complex with histones after nuclear transfer, acquiring a nucleoprotein structure analogous to that of native chromatin [23]. The use of the class 1 and 2 histone deacetylase (HDAC) inhibitor TSA increased pDNA-based seRNA expression in our experiments which at least suggests a chromatin-mediated limitation of seRNA plasmid transcription as also shown before [24,25,26].

However, it is probably more decisive that expression rates between mRNAs and pure IRES expression systems on the one hand and seRNA on the other cannot be directly compared. The reason for this is that in the first case, transfected RNAs are automatically and directly inducing translation [27,28]. In contrast, this is not the case for seRNAs. Since they are inactive in non-target cells, a series of steps must be completed in target cells before full activation and thus IRES-dependent translation can occur. In particular, this includes the stable establishment of a sense–antisense interaction [29], and, after partial degradation [7], the functional refolding of the IRES [30]. How many of the transcribed or transfected seRNA molecules complete these processes and thereby initiate the translation of the effector cannot be conclusively answered and requires further analysis. However, for many potential applications fields, low expression rates are even beneficial, since, e.g., cytotoxic protein expression in cancer therapy just depends on a low protein copy number while being largely dependent on cell type-specific expression. This is where seRNA shows its strengths.

From the perspective of the applicability of the seRNA technology, our analysis on the transferability of the selective expression of seRNAs is an important step. They show that different vector systems, such as AAV vectors or DNA minicircles, are indeed capable of further increasing the amount of the effector in target cells, while the leakiness of seRNA in non-target cells remains at a very low level. Even the highest amounts of AAV vectors with up to 50,000 seRNA particles per non-target cell are well tolerated and lead to transfection efficiencies and expression rates in target cells that are roughly equivalent to those of transfection with an mRNA expression plasmid (see Figure 4A). AAV-seRNA systems thus also allow the use of seRNA technology for applications that require a higher expression rate, such as in the treatment of genetic diseases [31]. The combination of a targeted delivery system [10] with target cell activation could offer a promising approach toward low dosing with high efficacy. Although not yet tested, based on these data, we also assume that other vector systems, such as circular [32] or self-replicating RNAs [33], may also play a functional role in the use of seRNA molecules.

A significant advancement over conventional seRNA plasmids is represented by IVT-seRNA, which exhibits a superior transfection efficiency and intensity [7]. IVT-seRNA has been demonstrated to offer substantially more precise control over expression in comparison to that facilitated by transcription from seRNA plasmids. Furthermore, the engineering of IVT-seRNA allows for a wider range of modifications, including the incorporation of modified nucleotides (see Supplementary Figure S5). Despite the ongoing challenges associated with the utilization of modified nucleotides in the IRES [34], the use of various cap analogs and Poly(A) modifications has emerged as a promising avenue for further exploration. Most importantly, the incorporation of translationally inactive AP3G cap structures facilitates a substantial advancement of seRNA, allowing the substitution of uORFs in the antisense of seRNA and therefore possibly also the significant shortening of the antisense domain. However, we are not trying here to rate the different delivery systems. We are demonstrating that the underlying cell type-specific activation mechanism remains fully functional regardless of the delivery system chosen. This provides scientists with a wide range of possibilities defined by the requirements of a chosen indication.

When comparing this technology with RADAR technology [35,36], which can also be used for cell type-specific expression, an increase in activity is achieved, in particular through the additional expression of the protein adenosine deaminase. However, this has so far led to a simultaneous, massive increase in off-target effects [35,36,37] for this technology. In contrast, we were able to show that seRNA technology is extremely robust against different approaches to increasing the expression of the encoded effector. It thus offers a wide range of adaptation options that can play an important role in future approaches, depending on the medical needs.

## Figures and Tables

**Table 1:** Probes for qRT-PCR.

Target Gene	Species
GAPDH, Hs02786624_g1, Thermo Fisher, Waltham, MA, USA	human
eGFP, Mr04097229_mr, Thermo Fisher, Waltham, MA, USA	non
IL6, Hs00174131, Thermo Fisher, Waltham, MA, USA	human
TNFα, Hs00174128, Thermo Fisher, Waltham, MA, USA	human
IL18, Hs01038788_m1, Thermo Fisher, Waltham, MA, USA	human
IL1β, Hs01555410_m1, Thermo Fisher, Waltham, MA, USA	human

## Data Availability

The original contributions presented in this study are included in the article/Supplementary Material. Further inquiries can be directed to the corresponding author.

## Supplementary Materials

The following supporting information can be downloaded at: https://www.mdpi.com/article/10.3390/pharmaceutics17121595/s1, Figure S1: seRNA activation mechanism compared to a conventional eGFP plasmid; Figure S2: Plasmid maps of the applied DNA constructs; Figure S3: Example of the purity of a IVT-seRNA-eGFP analyzed by denaturating 1% agarose gel electrophoresis; Figure S4: Example of transfected plasmids labelled with Cy5; Figure S5: Application of modified nucleotides in IVT-seRNA abolishes translational activity.
