# Supplementary material for: Robust Functionality and Regulation of Selectively Expressed RNA as AAV Vectors and In Vitro Transcribed Molecules
Source: Pharmaceutics. 2025 Dec 10;17(12):1595. doi: 10.3390/pharmaceutics17121595 (PMC12736599; doi:10.3390/pharmaceutics17121595)
Supplement: Supplementary file 1 [file pharmaceutics-17-01595-s001.zip › pharmaceutics-3912218-supplementary.pdf]

pharmaceutics

MDPI

Supplementary Materials

Robust Functionality and Regulation of Selectively Expressed RNA as AAV Vectors and In Vitro Transcribed Molecules

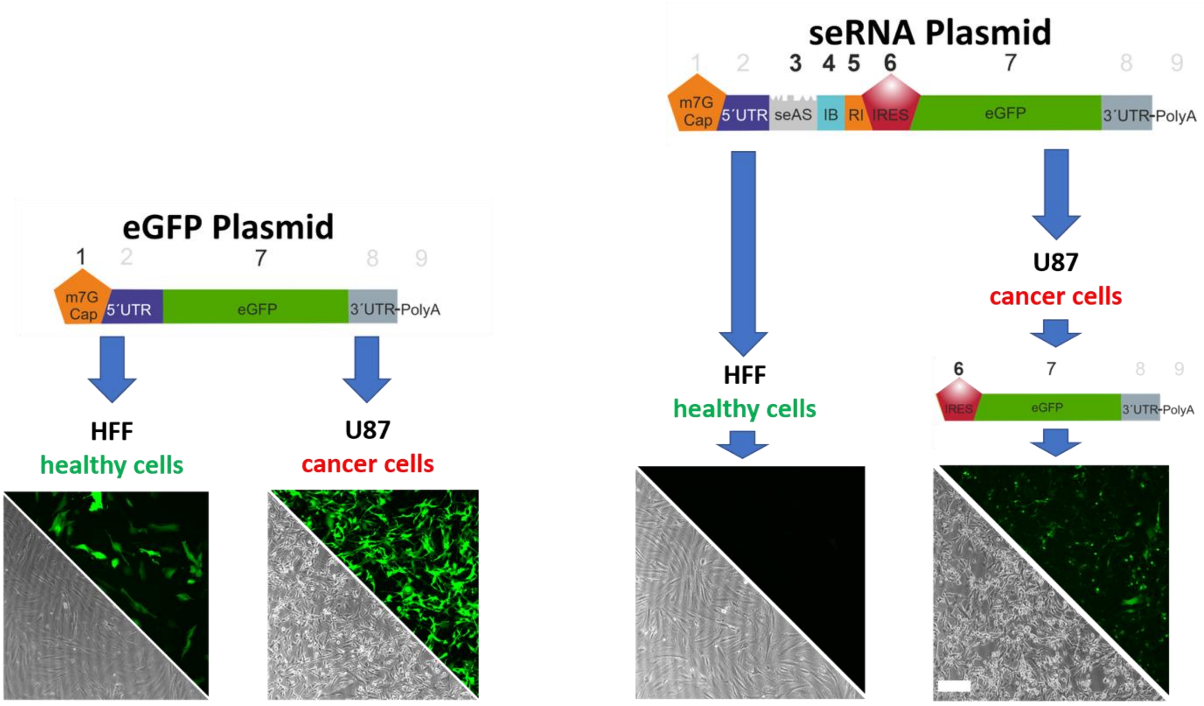

Figure S1. seRNA activation mechanism compared to a conventional eGFP plasmid. A conventional eGFP plasmid (left) is transcribed in every cell. The resulting capped (m7G Cap) mRNA is typically composed of an untranlated 5′-region (5′UTR), the coding sequence (eGFP), an untranslated 3′-region and a poly-A tail (3′UTR-PolyA) and translated independent on cell type (e.g. Human foreskin fibroblasts (HFF) or human glioblastoma cell line U87) to form the encoded protein. seRNA plasmid (right) is also transcribed in every cell. However, in healthy cells which do not express a certain target RNA, the seRNA stays inactive, because 5′-Cap-dependent expression is blocked by short upstream open reading frames (uORFs, located in the gray domain (seAS)). In addition, IRES-dependent expression is inhibited by a short IRES blocker (IB) that interferes with the functional IRES secondary structure.

In target cells as e.g. cancer cells, a cell-type specific target RNA binds to the antisense sequence (seAS). This double stranded RNA sequence is recognized by RNase H and RNase type III family members to induce seRNA degradation. However, 3′-directed degradation only removes the IRES blocker but is inhibited by a conserved viral RNase Inhibitor (RI) module. Removal of the IRES-blocker allows IRES functional refolding to express the encoded protein only in target cells. For a more detailed explanation of the seRNA functional mechanism see also [7].

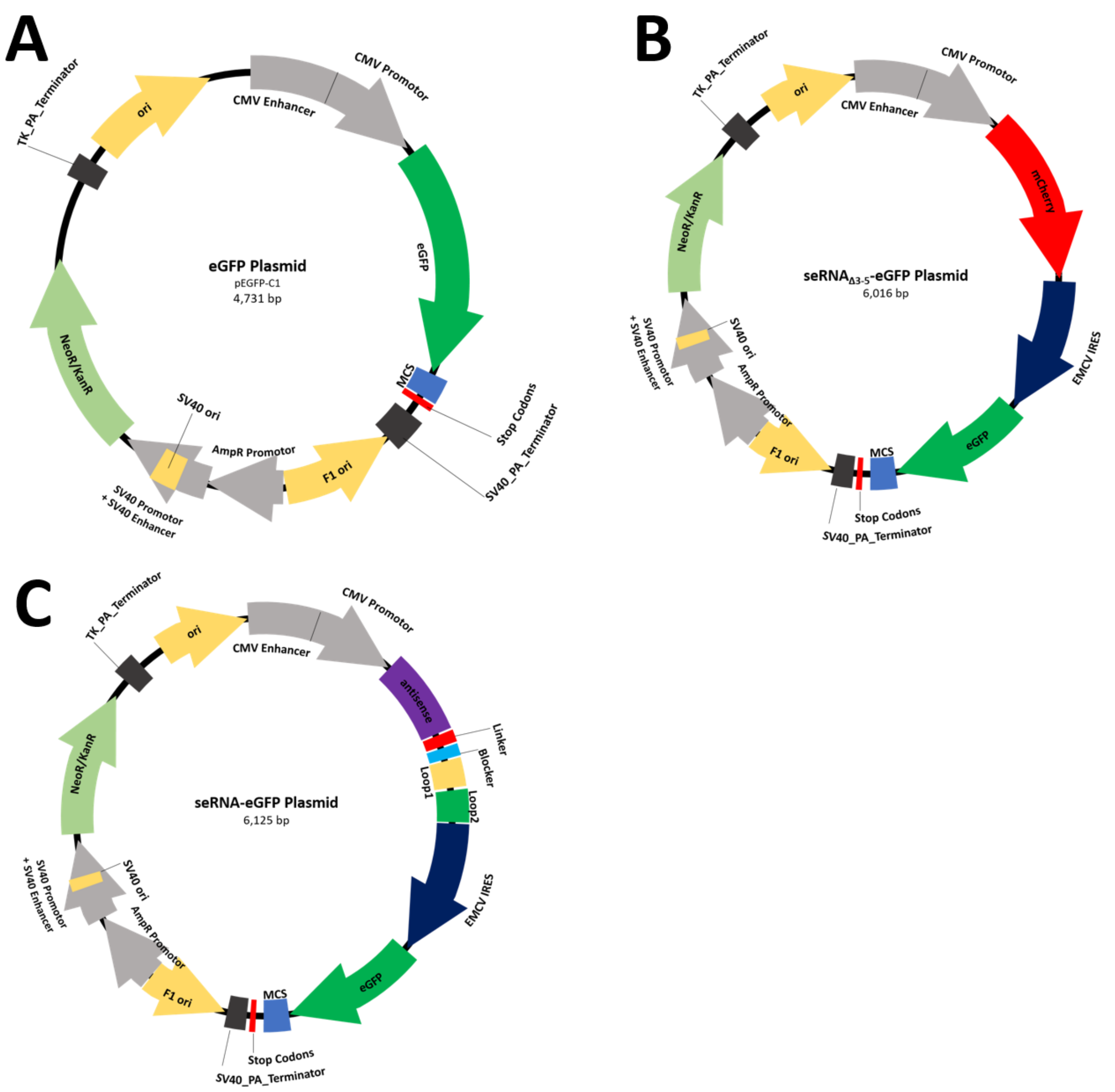

Figure S2. Plasmid maps of the applied DNA constructs. (A) eGFP Plasmids as conventional expression plasmid control. (B) seRNA$_{\Delta 3\text{-}5}$-eGFP as unselective control plasmid. (C) seRNA-eGFP full length construct.

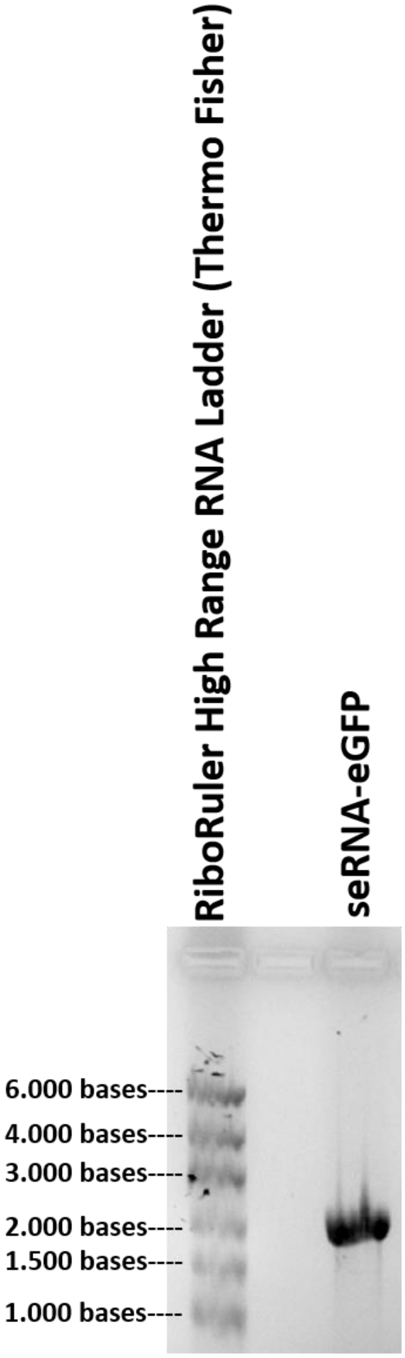

Figure S3. Example of the purity of a IVT-seRNA-eGFP analyzed by denaturating 1% agarose gel electrophoresis. As Ladder we used the RiboRuler High Range RNA Ladder (Thermo Fisher, Waltham, MA, USA).

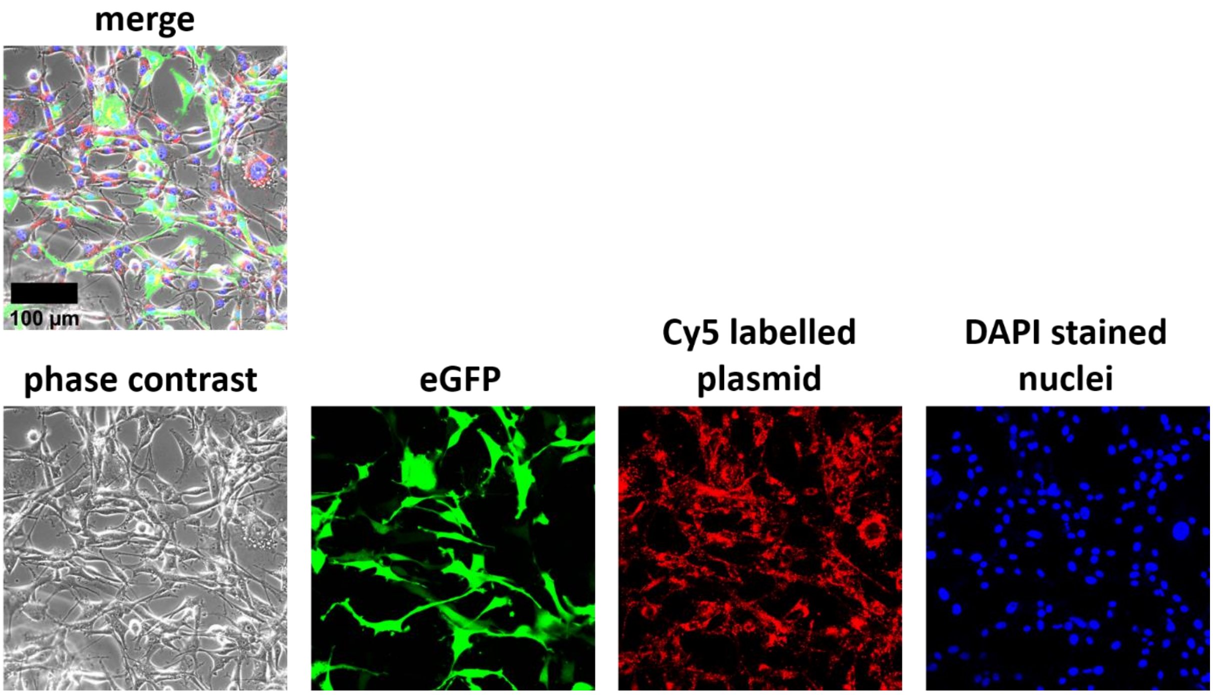

Figure S4. Example of transfected plasmids labelled with Cy5. The microscopy image shows U87 glioblastoma cells transfected with a eGFP-plasmid labelled with Cy5 fluorophores. The plasmid still expresses the eGFP protein it encodes. To detect the nuclei we stained them using a NucBlue Live ReadyProbes reagent (Thermo Fisher, Waltham, MA, USA).

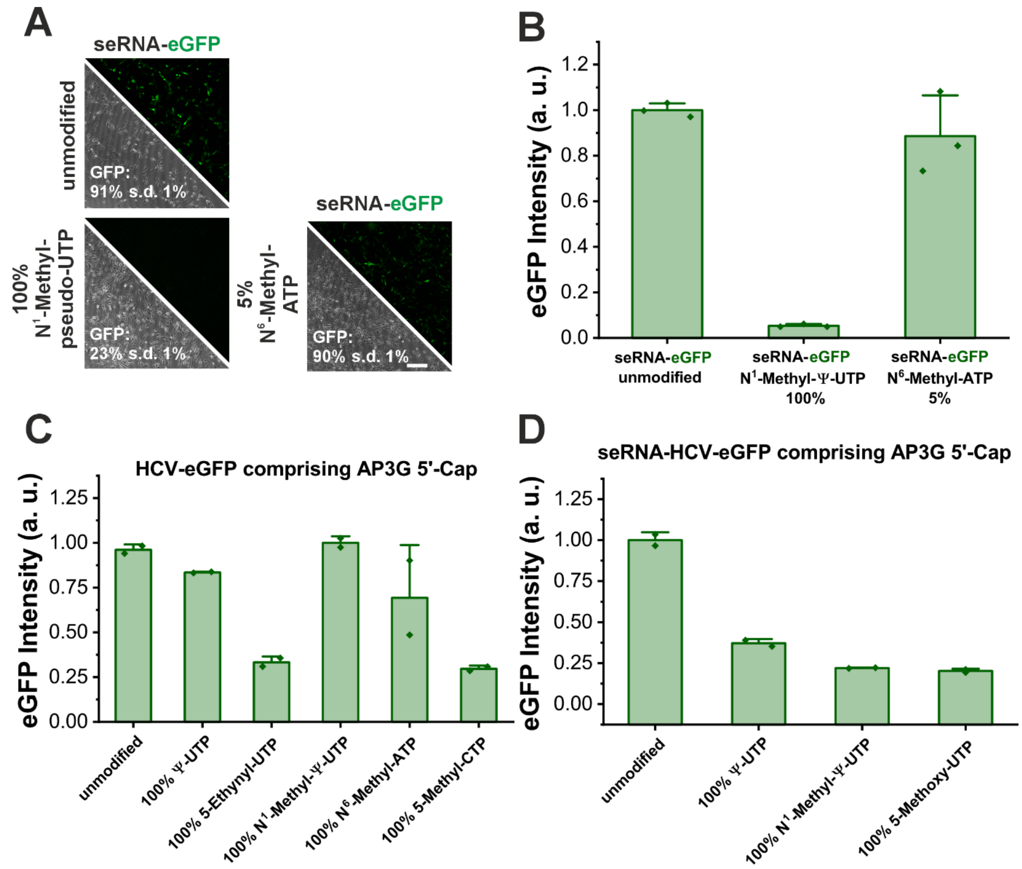

Figure S5. Application of modified nucleotides in IVT-seRNA abolishes translational activity. (**A**) Microscopic visualization and expression levels (**B**) of the seRNA-eGFP construct containing the EMCV IRES in U87 glioblastoma cells. The types of modified nucleotides used are indicated in the figure; transfection efficiency is shown with standard deviation (s. d.). n = 3 independent samples.

Scale bar: 200 μm, (**C**) eGFP expression intensity of a non-selective HCV-eGFP control comprising a 5′-AP3G cap analog. Different modified nucleotides were tested, as indicated in the figure. n = 2 independent samples. (**D**) eGFP expression intensity of an HCV-containing seRNA-eGFP construct comprising an AP3G cap. Different modified nucleotides were tested, as indicated in the figure. n = 2 independent samples. All modified nucleotides were obtained from Jena Biosciences, except for 5-ethynyl-UTP (Baseclick). The concentrations used corresponded to those applied for unmodified nucleotides.
